# Deep antiquity of seagrasses supporting European eel fisheries in the western Baltic

**DOI:** 10.1098/rspb.2024.0674

**Published:** 2024-07-24

**Authors:** Eric Guiry, Harry K. Robson

**Affiliations:** ^1^ Department of Anthropology, Trent University, 1600 Westbank Drive, Peterborough, Ontario, Canada K9L 0G2; ^2^ School of Archaeology and Ancient History, University of Leicester, Mayor's Walk, Leicester LE1 7RH, UK; ^3^ BioArCh, Department of Archaeology, University of York, Heslington, York YO10 5DD, UK

**Keywords:** European eel, western Baltic, stable isotope analysis, seagrass meadows, historical ecology

## Abstract

Protecting ocean habitats is critical for international efforts to mitigate climate impacts and ensure food security, but the ecological data upon which policy makers base conservation and restoration targets often reflect ecosystems that have already been deeply impacted by anthropogenic change. The archaeological record is a biomolecular archive offering a temporal scope that cannot be gathered from historical records or contemporary fieldwork. Insights from biogeochemical and osteometric analyses of fish bones, combined with context from contemporary field studies, show how prehistoric fisheries in the western Baltic relied on seagrass meadows. European eels (*Anguilla anguilla*) harvested by Mesolithic and Neolithic peoples over millennia showed a strong fidelity for eelgrass foraging habitats, an ecological relationship that remains largely overlooked today, demonstrating the value of protecting these habitats. These data open new windows onto ecosystem- and species-level behaviours, highlighting the need for wider incorporation of archaeological data in strategies for protecting our oceans.

## 1. Introduction

Marine environments are in peril [1,2]. While marine and estuarine resources have been harvested in European waters for millennia [3,4], the points at which this exploitation and other environmental impacts became unsustainable remain poorly understood [5]. With species and ecosystems in precipitous decline in many areas, policy makers need evidence-based strategies for restoration, but a dearth of reliable ecological baselines for contextualizing management decisions remains a major obstacle [6,7]. In particular, and in the context of long-term human impacts, the relatively recent advent of scientific monitoring (typically dating only to the twentieth century) means that most available ecological baselines represent ecosystems that were already profoundly altered by industrialized human activities [1]. Alternative lines of retrospective evidence can provide solutions, including interdisciplinary engagement with the archaeological and palaeontological records, which preserve physical and biomolecular records of past biodiversity and ecosystem processes [8]. For instance, studies combining zooarchaeological and isotopic approaches have recently uncovered undocumented overfishing events and previously invisible anthropogenic impacts on behavioural ecology [9–11]. Considering the vast wealth of such materials available in the collective European archaeological record, these approaches are still, comparatively speaking, astonishingly rare and, moreover, are typically not explicitly framed within an ecological perspective, leaving much of this potential untapped.

With respect to declines in European marine ecosystems and fisheries, some habitats and species have become foci for conservation efforts owing to their charismatic nature and broad economic importance. Among the most iconic of these are Europe's coastal seagrass (specifically eelgrasses, *Zostera* spp.) meadows and the once-ubiquitous European eel (*Anguilla anguilla*), which are now considered Threatened [12] and Critically Endangered [13], respectively. In addition to their deeply rooted historical roles—both significant sources of raw materials, food and symbolism in European culture—seagrasses (as a major carbon sink) and eels (as a fishery resource) have roles to play in addressing ongoing sustainability issues, including climate change and food security [14,15]. Despite these contributions, we know little about their respective long-term stability prior to the twentieth century. For instance, while seagrasses have long been used as a building material [16] and are thought to be a key source of Blue Carbon storage [17], their role in supporting human ecosystems prior to steep declines by the early 1900s remains undocumented [18]. Likewise, while European eel populations have been decimated (a 98% decline) since 1980 [19], eels have long been harvested as a cosmopolitan, highly desirable food species [20]. In that context, and given their distribution across much of Europe, restoration of this species holds potential to improve fisheries resources for hundreds of millions of people [21]. In both cases, however, while archaeology tells us that eels and eelgrass habitats have been critical for Europeans, limited work has been done to establish a framework and baseline for contextualizing the longer-term ecology of their use (although see [4,22]). This dearth of data has limited the rich potential for archaeology to offer retrospective analyses valued for informing policy.

**Figure 1.**
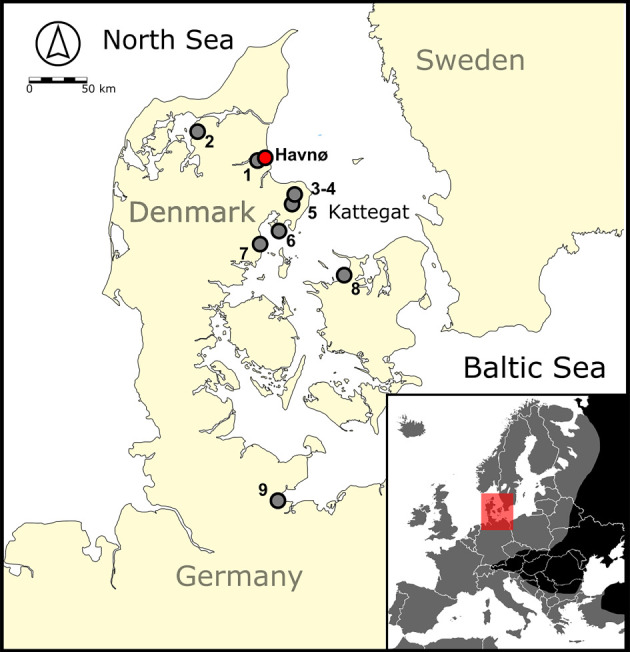
Map showing the locations of Havnø (red symbol) and other archaeological sites (grey symbols) with isotopic evidence for harvesting fish that foraged intensively in eelgrass ecosystems (see §5a 'General dietary trends'; electronic supplementary material, table S1). Sites are numbered as follows: 1. Thygeslund, 2. Bjørnsholm, 3. Kainsbakke, 4. Kirial Bro, 5. Nederst, 6. Vængesø III, 7. Norsminde, 8. Dragsholm, 9. Neustadt. Inset map: grey shading shows range for wild eels in Europe; red shading shows study area.

We performed isotopic (*δ*^13^C, *δ*^15^N) and osteometric (size estimation) analyses of archaeological eel bones from the western Baltic to better understand connections between eels, eelgrass habitats and people in the past. Analyses aimed to address three research questions: (1) To what extent were past eel populations utilizing marine habitats?; (2) Is there evidence for eelgrass habitat specialization?; (3) How do these archaeological perspectives compare with contemporary isotope-based narratives about eel behaviour? Results indicate that eelgrasses provided a preferred habitat for eels caught and consumed by Late Mesolithic and Early–Middle Neolithic (*ca* 5400 to 2550 cal Before Common Era (BCE)) peoples in the western Baltic (figure 1). This long-term connection between eels and seagrass habitats provides the first evidence for the deep antiquity of eelgrass ecosystems supporting human societies in Northern Europe and underscores the importance of seagrass habitat restoration for the sustainability of European fisheries.

## 2. Ecological, historical and archaeological context

European eels have a complex catadromous lifecycle, involving iconic migrations of up to 8000 km across inland waters, seas and the Atlantic Ocean (for review, see [23,24]). Adult eels migrate from Europe to the Sargasso Sea, a region of the Caribbean, where they reproduce. There, eggs hatch to become leptocephalus larvae, which, while drifting on the Gulfstream back towards Europe over the course of about 2 years, develop to become glass eels. From here they develop into elvers and then yellow eels, the main and longest life stage in which they grow and mature into adults. Glass and yellow eels move from the ocean to coastal waters or upriver into freshwater habitats, where they are highly adaptable in terms of both their habitat use and diet [23]. Between the ages of 5 and 20 (and sometimes longer in the Baltic), adults transition to silver eels, their final life stage, preparing them for their return migration to the Sargasso Sea.

Archaeological evidence for eel use is common across much of Europe, with some finds documenting eel use tens of thousands of years before present [4]. Concentrations of exploitation appear in Northern and Western Europe during the Mesolithic and Neolithic periods (for review, see [4]). Technologies for early eel collection involved spears, including barbed points, harpoons and leisters as well as hooks, nets, traps and weirs [22]. Throughout Europe, in areas where eels found productive habitat, humans have developed increasingly sophisticated ways of collecting more of them more efficiently. Increasing modification of wetlands, rivers and coastal environments has further impacted eels by destroying, polluting, or blocking access to key habitats [25–27]. By at least the 1800s, exploitation and habitat loss had intensified across many of Europe's former eel strongholds to a level that began to impact the number of adults that could return to spawn [20,26]. For instance, specialized dams built across some of Europe's most productive deltas and inland habitats, which take advantage of the silver eel's migratory behaviour, trap a large fraction of outmigration [28]. Today, eels remain a major source of revenue and traditional food for many countries and the EU's management decisions aim to balance the deep cultural significance of eels as food with strategies to promote their restoration [29].

## 3. Methods

### (a) Experimental design and sample selection

We analysed the isotopic compositions of collagen extracted from archaeological eel bones. Compared with tissues that are most frequently analysed for contemporary ecological research (e.g. skin, muscle), isotopic compositions of bone collagen offer a longer-term perspective on diet and habitat use [30]. Because bone collagen remodels slowly over the life of an organism, its isotopic composition will reflect a multi-year average of consumed foods. This longer-term averaging means that bone collagen isotopic compositions from longer-lived animals, like eels, will not be as strongly influenced by seasonal or other short-term dietary variations and are therefore suitable for exploring lifetime-scale patterns in behaviour.

Specimens were selected from a column sample spanning well stratified deposits at the Havnø shell midden, located on the north shore of the Mariager Fjord in east-central Jutland, Denmark (figure 1). Based on radiocarbon and seriation-based dating of artefacts (see [31]), the sampled eel remains date primarily to the Late Mesolithic Ertebølle culture (5250–4000 cal BCE). For full discussion of contextual details and dating, see electronic supplementary material, text S1 and table S2.

To obtain a larger dataset for this study, we sampled widely across skeletal elements in each stratigraphic level (e.g. by including multiple vertebrae from the same context). While this means that in some cases we may have sampled the same individual more than once, we note that within each stratigraphic level a range of reconstructed sizes (total length (TL) estimated in centimetres) and isotopic variation (differences greater than 1.5‰ expected for typical intra-skeletal isotopic variation; [32]) occurs. This suggests that instances of sampling the same individual multiple times were uncommon.

### (b) Isotopic analyses

Owing to very small sample sizes available from most specimens (with starting masses as small as 1.5–2.0 mg), collagen extractions were carefully monitored to maximize collagen recovery while following established protocols outlined in electronic supplementary material, text S2 and tables S3–S6. Isotopic compositions were measured on 0.5 mg subsamples of collagen using an EA-IRMS at the Archaeology Chemistry Laboratory at the University of British Columbia. For *δ*^13^C and *δ*^15^N standard uncertainties were ±0.11 and ±0.22‰, respectively. Full isotopic calibration and quality assurance protocols are outlined in electronic supplementary material, text S3. Integrity of the isotopic data was evaluated using carbon (greater than 13.8%) and nitrogen (greater than 4.0%) elemental concentrations [33] and liberal C : N quality control (QC) criteria developed for cold-water fish [34], which allows for a *δ*^13^C shift of up to –1.0‰ due to humic acid contamination.

### (c) Statistics

Correlation analyses (Pearson's *r* and Spearman's *ρ* for TL versus both *δ*^15^N and *δ*^13^C to evaluate the impact of size-linked trophic level shifts on isotopic variation), comparison of mean *δ*^13^C for temporal groups (Mesolithic versus Neolithic), and a generalized extreme studentized deviate (GESD) test for identifying outliers were performed using PAST, version 4.13 (electronic supplementary material, text S4). Group mean comparison for *δ*^15^N was not performed because variation among these data are driven by TL-dependent trophic position, and sample sizes for individual reconstructed TL size category groups were too small for statistically meaningful comparisons.

### (d) Osteology

Taxonomic identifications were assigned following standard principles of comparative osteology (see [31]) using the fish skeletal reference collection at the BioArCh research centre at the University of York. Estimation of eel TL was accomplished through side-by-side comparison with a catalogue of modern eel skeletons spanning a range of sizes identified in regional archaeological records. Specimens were assigned to a 5 cm TL bin increment based on the modern specimen that they matched most closely. Although regression formulae are often used to reconstruct archaeological fish length (based on measurements between specific bone landmarks), our size-matching approach, using direct comparisons with known-size modern specimens, allowed us to assign more archaeological specimens to a TL bin. This was particularly important for fragmented specimens that lacked landmarks chosen for regression formulae.

## 4. Results

Most samples (87%, *n* = 65) passed liberal QC criteria designed for cold-water fish [34] (electronic supplementary material, table S2). Additional previously published eel *δ*^13^C and *δ*^15^N data from Havnø include eight samples (passing QC), as well as *δ*^34^S values from two samples [31,35] (electronic supplementary material, table S2). For *δ*^13^C and *δ*^15^N data, GESD tests found no significant outliners (no *R*_crit_ where *p* < 0.05). Together, eels (*n* = 74) produced a wide range of variation in *δ*^13^C and *δ*^15^N, spanning 9.1‰ (from –13.5 to –4.4‰, mean = –8.1 ± 1.8‰) and 5.9‰ (from +5.0 to +10.9‰, mean = +7.9 ± 1.2‰), respectively. Two samples have *δ*^34^S values of +5.7 and +7.2‰. Compared with published bone collagen isotopic compositions from other Early–Middle Holocene archaeological fish from Europe (*n* = 427, collated from 44 publications; electronic supplementary material, table S1), our eel samples occupy the high end of this pan-European *δ*^13^C spectrum. TL reconstructions were possible for 69 samples with isotopic compositions passing QC (electronic supplementary material, table S2), and span 20–75 cm with an average sample-wide TL of approximately 39 cm, indicating that many of these individuals were harvested as yellow eels. TL shows strong positive correlations between *δ*^15^N (Pearson's *r* = + 0.441, *p* ≤ 0.001; Spearman's *ρ* = 0.458, *p* < 0.001; electronic supplementary material, figure S1) but not *δ*^13^C (Pearson's *r* = –0.029, *p* = 0.815, Spearman's *ρ* = –0.026, *p* = 0.830; electronic supplementary material, figure S2). As comparison of group means for *δ*^13^C dating to the Mesolithic (*n* = 51; Shapiro-Wilk *W* = 0.974, *p* = 0.315) versus the Neolithic (*n* = 11; Shapiro-Wilk *W* = 0.971, *p* = 0.900) periods showed no significant difference (Levene's test, *p* = 0.589; Student's *t* = 0.693, d.f. = 612, *p* = 0.490), we did not further assess temporal dimensions of these data, but note that chronological variation in European eel isotopic compositions has been explored previously [35].

## 5. Discussion

### (a) General dietary trends

Our results add to a growing body of isotopic evidence documenting anguillid habitat flexibility (e.g. [35–39]). For eels analysed in this study (all yellow or silver eels based on size), we expect that the isotopic compositions of bone collagen (which remodels slowly over long periods of time) will reflect diet over several years of life. Isotopic research has played an important role in complementing observational studies by helping to quantify the importance of different habitats. These archaeological eel *δ*^13^C, *δ*^15^N and *δ*^34^S compositions advance our understanding in mutually reinforcing ways.

**Figure 2.**
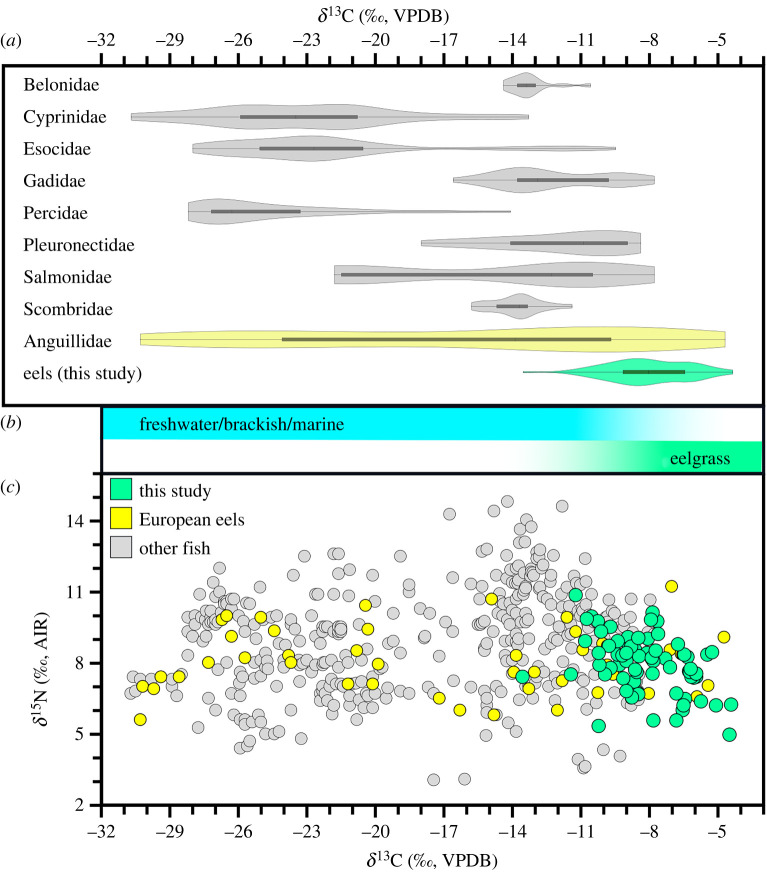
Bone collagen isotopic compositions from eels and other European Early–Middle Holocene archaeological fish (all data presented in electronic supplementary material, tables S1 and S2). (*a*) Violin and box plots showing kernel densities of *δ*^13^C for Havnø eels (green), eels from other sites (yellow), and other common fish taxa grouped by family (with 15 or more published isotopic compositions meeting QC criteria; see electronic supplementary material, table S1). (*b*) Schematic representation of *δ*^13^C ranges expected for bone collagen of consumers of eelgrass relative to other kinds of aquatic primary production (for review, see [40]). (*c*) Isotopic composition of Havnø eels (green symbols) and previously published eel (yellow symbols) and other fish (grey symbols). AIR=air (standard for reporting *δ*^15^N values). VPDB=Vienna Pee Dee Belemnite (standard for reporting *δ*^13^C).

Eel *δ*^13^C falls at the extreme end of the spectrum for all contemporaneous as well as earlier and later (Early-Middle Holocene) European fish bone collagen isotopic compositions (figure 2) and this provides an opportunity to offer clearly resolved interpretations for intensive use of specific habitats. Stable carbon isotope compositions in aquatic environments are highly variable at both spatial and temporal scales, being contingent on seasonal and physical factors (bathymetric, flow, thermoclines etc.) governed by carbon sources and cycling [40]. A key source of *δ*^13^C variation across aquatic habitats occurs prior to carbon entering the food web. Specifically, the dissolved inorganic carbon (DIC) pools available to marine primary producers for photosynthesis are typically enriched in ^13^C relative to those in freshwater habitats [40,41], leading to widespread expectations that marine versus freshwater residency can be distinguished based on variation in *δ*^13^C (e.g. [42,43]). In that context, while it has often been assumed that freshwater food webs will have lower *δ*^13^C than their oceanic counterparts, they can have *δ*^13^C baselines that overlap to a large extent with food webs in marine habitats [40]. This means that, particularly for behaviourally flexible taxa, baseline data are often essential for interpreting fish behaviour based on isotopic compositions [44]. For instance, archaeological American eel (*Anguilla rostrata*) bone collagen *δ*^13^C values spanning –27.7 to –16.1‰ have been observed for adults in distant inland freshwater locations, including Lake Ontario [36], where no appreciable residual isotopic signature from their earlier marine life stages are expected [40]. Moreover, observed freshwater fish *δ*^13^C values as high as –11.5‰ occur in that same study region, indicating an extreme freshwater endpoint overlapping with most of the observed range for marine species bone collagen *δ*^13^C (figure 2). For these reasons, establishing marine residency based on fish isotopic compositions requires close consideration of how relevant local carbon sources and cycling processes govern isotopic variation [44].

As a source of primary production, seagrass habitats appear to be an exception because they routinely occupy an endpoint position for high *δ*^13^C among primary production in marine habitats. For this reason, consumers in food webs supported by seagrass meadows appear further enriched in ^13^C relative to their marine, non-seagrass-inhabiting counterparts [45]. Higher *δ*^13^C values in seagrass primary production is thought to reflect slower CO_2_ diffusion rates (which reduces photosynthetic discrimination against ^13^C) and the capacity for seagrasses to assimilate HCO_3_^−^ (which is ^13^C-enriched relative to the primary DIC source, CO_2_) (for review, see [46]). This creates a zone at the highest end of the observed *δ*^13^C spectrum for aquatic consumer bone collagen (figure 2*b*) that is dominated by animals that use eelgrass (or other seagrass) habitats, and which falls beyond the observed range for other marine and freshwater biota. For instance, recent studies focusing on herbivores specializing in seagrass habitats observed consumer *δ*^13^C values that extend the higher-end boundaries for the known range for bone collagen of archaeological marine animals. Specifically, sea turtle bone collagen from the tropics had *δ*^13^C values up to *ca* –3.0‰ ([47] and up to –5.0‰ from the Mediterranean [48]), though latitudinal impacts on the *δ*^13^C of marine DIC [49] mean that an endpoint in temperate locations, such as our study region, would be 2–3‰ lower. In that context, while we therefore expect a degree of overlap in the potential *δ*^13^C range for bone collagen from consumers living in marine, estuarine and freshwater habitats (from the bottom end of the marine *δ*^13^C range up to about –9.0‰), values falling well above this threshold result from intensive use of seagrass habitats. Most of our results fall above this range. This indicates that, while European eels inhabiting the study region in the archaeological past almost certainly had access to a wide range of habitats spanning the freshwater–marine spectrum, most of the eels we have analysed foraged in prehistoric eelgrasses meadows in the western Baltic. It is worth noting that, although we strongly suspect based on intra-context size and isotopic variation (see Methods) that most of our samples represent unique individuals (i.e. different eels), uncertainty remains about how many eels are represented in our sample. However, we note that extremely high *δ*^13^C values (>–9.0‰) are present in all nine sampled archaeological contexts, which means that even if we took the most conservative possible estimate of how many eels are represented by our sample (i.e. 1 per context), these data still demonstrate a long-term presence of eels that intensively foraged in eelgrass meadows.

Eel *δ*^15^N values support these *δ*^13^C-based interpretations. Even though most of our eel samples exhibit *δ*^13^C patterns strongly influenced by the incorporation of eelgrass-derived nutrients, it remains possible that some of the *δ*^13^C variation we have observed (specimens with *δ*^13^C values at the lower end of our sample range) could come from individuals that migrated between and used resources in both freshwater and marine habitats. If this were the case, however, we would expect to see considerable variation in *δ*^15^N. This is because *δ*^15^N baselines can differ dramatically between habitats [43] owing to prevailing differences in the dominant dissolved inorganic nitrogen sources and cycling processes governing *δ*^15^N in marine and freshwater ecosystems [40,50]. Indeed, *δ*^15^N values have been observed to differ systematically between allopatric European eel populations living at different points along salinity gradients [37–39]. Our data do not show this. By contrast, while we have observed considerable *δ*^15^N variation, a strong positive correlation between estimated TL and *δ*^15^N (electronic supplementary material, figure S1) indicates that this variation reflects size-driven trophic level increases. In other words, this is a strong indication that the variation in *δ*^15^N is due to size-dependent prey choice (i.e. trophic level; [51]) rather than movement between areas with drastically differing isotopic baselines. In that context, these data are consistent with our interpretation that all of the eels we have analysed, even those with lower *δ*^13^C values, used the same marine habitat, focusing on eelgrass beds.

Stable sulfur isotope values from two samples support the interpretation that extreme eel *δ*^13^C values reflect a long-term, intensive focus on eelgrass habitats. While marine environments are thought to have a globally homogeneous sulfate isotopic composition, with a high *δ*^34^S of approximately +20.0‰ [52], there are contexts in which this broad-scale observation does not hold [47,53,54], and seagrass habitat use offers one important exception (for review see [55,56]). While most marine primary producers (and their consumers) have *δ*^34^S values close to those of marine sulfates, eelgrasses can incorporate sulfide-cycled sulfur [57,58]. Reduction of sulfate to sulfide in anoxic sediments is associated with a strong discrimination against ^34^S [59,60], meaning that when associated sulfur is eventually incorporated into marine food webs it imparts distinctively low *δ*^34^S values in local consumers (e.g. [47,53,61,62]). Havnø eel *δ*^34^S values are much lower than would be expected for typical marine consumers, which is consistent with the interpretation that these eels relied on primary production derived from eelgrass meadows.

It is important to bear in mind that a common archaeological (and occasionally ecological) expectation, that estuarine environments will have lower *δ*^34^S reflecting inputs from freshwater sulfates [63], is likely not realistic. A large study that systematically explored *δ*^34^S variation among fish communities across a freshwater-to-marine salinity gradient showed that even at salinity levels as low as 0.6 ppt marine-derived sulfur dominated sulfate and consumer *δ*^34^S values [64]. This makes sense on the basis of mass balance, as marine environments are at least an order of magnitude richer in sulfates than typical freshwater environments. Therefore, interpreting these lower eel *δ*^34^S values simply as evidence for freshwater influence on estuarine feeding does not offer a more plausible explanation.

In sum, while these interpretations are based primarily on the extreme nature of *δ*^13^C values in our sample, a cohesive trophic framework, as evidenced by TL versus *δ*^15^N comparisons, and use of *δ*^34^S as an independent biogeochemical indicator, offer supporting evidence. In this context, data from the small number of eels in our sample with less extreme *δ*^13^C values (i.e. *ca* <–9.0‰) are also consistent with use of eelgrass habitats. This is because eelgrasses provide a platform for epiphytic algae, which offer a primary production source with lower *δ*^13^C values (see below). Indeed, contemporary ecological studies of animals foraging in eelgrass habitats show that consumers using these areas can have much lower *δ*^13^C values in cases where the carbon in food is ultimately sourced from epiphytic primary production [65,66]. Taken together, these three lines of evidence show that, at least in the past, the European eel population included communities with a sustained habitat preference for eelgrass meadows. While this behaviour has yet to be identified isotopically in contemporary eel populations (e.g. [37–39]), these results indicate that a strong preference for seagrass habitat falls within the behavioural envelope of the European eel population.

Furthermore, considering the broader dataset of published archaeological fish bone collagen isotopic compositions for Early–Middle Holocene Europe (figure 2), there is further evidence for European eel use of eelgrass habitats. While a large proportion of published eel data have lower *δ*^13^C values (*n* = 38 of 49 eels passing QC, or 78%, had *δ*^13^C values below –9.0‰, see figure 2*c*), consistent with use of diverse freshwater and marine habitats, eel samples (*n* = 16 from seven additional sites in the western Baltic, including: Dragsholm, *n* = 1; Kainsbakke, *n* = 8; Kirial Bro, *n* = 1; Nederst, *n* = 2; Neustadt, *n* = 1; Norsminde, *n* = 1; and Thygeslund, *n* = 2; figures 1 and 2*c*) with dates spanning approximately 5470 to 2550 cal BCE had *δ*^13^C values (electronic supplementary material, table S1) indicative of a strong eelgrass emphasis. These observations add considerable temporal depth to our interpretations, indicating that prehistoric European eels intensively used eelgrass meadows in the western Baltic for nearly 3000 years. In that context, it is worth noting that the archaeological literature for this region includes limited evidence (*n* = 18 samples; electronic supplementary material, table S1; including an additional two sites, Bjørnsholm and Vængesø III, figure 1) suggesting other taxa also used eelgrass habitats, including members of the Gadidae (*n* = 5), Pleuronectidae (*n* = 9) and Salmonidae (*n* = 4) families, highlighting the diversity of ancient fish with behavioural specializations relying on seagrass primary production.

### (b) Archaeological and anthropological relevance

Isotopic analysis (*δ*^13^C, *δ*^15^N, *δ*^34^S) of bone collagen from human remains has been widely adopted by archaeologists as a means of assessing the nature and evolution of human diet. This approach requires nuanced consideration of both a global set of isotopic ecological principles (a unifying framework connecting drivers of isotopic variation in human food webs) and region-specific variables (as evidenced through local baseline data). In that context, our data, highlighting how seagrass ecosystems have supported human subsistence in the past, have important implications for how these principles and variables are considered when interpreting human isotopic compositions in palaeodietary research.

Archaeological literature often assumes that humans with marine-intensive diets will have higher bone collagen *δ*^34^S values, reflecting the isotopic composition of seawater sulfate (which is typically expected to be higher than terrestrial foods; [63]). However, recent studies exploring isotopic compositions of marine fauna, including turtles [47], fish [53] and mammals [54], have highlighted how the intensive use of seagrass- and benthic microalgae-rich habitats can create isotopic patterns that would not be consistent with prevailing interpretive frameworks. Our data linking low *δ*^34^S values with extremely high *δ*^13^C values in eels confirm that these wider patterns, showing lower *δ*^34^S in other archaeological marine consumers elsewhere, occur in Northern Europe. Given the wide distribution of eelgrasses around coastal Europe [18,67], and the fact that seagrasses can be an important habitat for a wide range of economically significant marine species [68,69], this has implications for interpreting human diets across a broad geographical area. In our regional context, the western Baltic, archaeologists have struggled to explain how humans and other consumers with *δ*^13^C and *δ*^15^N values consistent with marine-focused diets can produce relatively low *δ*^34^S values [70] not consistent with the expected baseline for marine sulfate. In that context, these results provide a unified interpretive framework accommodating these previously dissonant interpretations. We recommend further *δ*^34^S work on a taxonomically diverse range of fish bone collagen to improve this interpretive framework.

Lastly, it would appear that the near 3000 year long use of eelgrasses meadows by European eels was not affected by anthropogenic or environmental impacts despite human migrations and population turnovers associated with cultural transitions during this period, and changes in the local vegetation, including deforestation [71].

### (c) Ecological and conservation relevance

Developing a better understanding of the nature and extent of marine habitat use by eels is important for fisheries conservation, particularly in the context of the growing impacts of climate change [20,24]. Owing to the European eel's complex lifecycle, we understand relatively little about its presence and behaviour in areas outside of freshwater and estuarine habitats [24]. In fact, it was only in 1997 that the first concrete evidence for the eel's capacity to complete its entire lifecycle without use of freshwater or brackish habitats was published [72,73]. A comprehensive review of the literature published since then highlights how marine residency in eels remains chronically understudied, with fewer than 50 publications on the topic [24]. In part this is due to challenges in recording eels in marine areas and confirming marine residency (presently the best methods are lethal to fish), but it is also at least partly related to lingering assumptions about eel catadromy [24]. This shortfall in research and monitoring has meant that management, which is primarily based on data from freshwater residents, overlooks marine eels [24]. In that context, basic details—about the size of marine resident eel populations, the specific habitats they use, the factors that promote marine residency behaviour, and the contribution marine residents make to overall recruitment—remain largely unevidenced [24]. These details are the building blocks for developing policy responses to managing this fishery resource and are therefore a research priority.

Broad contours of the geographical and climatic structuring of marine residency behaviour among European eels have begun to emerge. There is evidence that marine residency is more prevalent in the northern and southern ends of the eel's range, where physical and physiological barriers to enter freshwater are greatest [74–76]. This is linked to temperature at multiple levels and has led to hypotheses about temporal trends in the prevalence of marine residency behaviour [24]. For instance, it could suggest that marine residency has increased over recent years as a plastic response to increasing variability in the utility of freshwater environments for eels. Alternatively, it could be that we have only recently recognized what has always been an important behavioural component of the European eel population. While these hypotheses are not mutually exclusive, our data provide concrete evidence that the latter explanation has been the case since at least *ca* 5470 cal BCE. In other words, it is now abundantly clear that marine residency among European eels has a deep history (at least in the western Baltic) and, moreover, was characteristic of at least some components of the eel population before the advent and intensification of industrial-scale exploitation over the last few centuries, which coincided with the species' precipitous decline [20]. While further analyses of archaeological eel specimens, aimed at exploring the geographical extent of prehistoric marine residency, would help to contextualize these findings, the occurrence or marine residency in the distant past and today (following mass declines) suggests that the presence of this behaviour is not mediated by intensity of exploitation.

If comparatively little is known about marine residency among European eels, still less is known about their affinity for eelgrass and other seagrass habitats. While the American and European eels’ presence has long been documented in or near eelgrass meadows [77–81], the extent to which they specialize in foraging in these habitats remains a mystery. In part this is because it is difficult to assess long-term residency in any habitat, given that some individuals practise habitat shifting as adults [74,75,82] and may simply be passing through an area when encountered during monitoring programmes. One study found that American eel abundance was higher in areas where eelgrass was in decline compared with areas where eelgrass habitats were intact [79]. By contrast, another study, investigating interspecific competition between eels and Lusitanian toadfish (*Halobatrachus didactylus*) towards the southern end of the European eel's range, found that within marine and estuarine environments substantial concentrations of adult eels were located in eelgrass habitats [83]. However, interpretations of the extent to which these results reflect an eel preference for this habitat were complicated by the study's design, which focused on interspecific competitive exclusion and/or predator avoidance across habitat types. This left open the possibility that the observed affinity for eelgrass habitats reflected local conditions and ecosystem dynamics rather than a potential species-wide habitat specialization. Our results offer new insights confirming that a component of the eel population can, and for millennia did, consistently focus their foraging on eelgrass habitats. This highlights the value of these habitats and moreover, provides evidence that, among marine resident eels, habitat selectivity strongly influences foraging behaviour. While both of these insights underscore the importance of efforts to restore seagrass habitats more generally [17,18,84], they also offer a unique window onto how seagrass ecosystems have functioned in the past, information that may have valuable conservation implications.

Although we have shown that isotopic composition of our eel sample firmly links them to seagrass habitat use, the extreme *δ*^13^C values produced by some samples (up to *ca* –4.0‰) highlight a particularly strong degree of connectivity between eelgrass, as a primary producer and higher trophic levels. Isotopic studies of consumers that use seagrass habitats have noted differences between seagrasses versus algal primary production (including epiphytic algae growing directly on seagrasses), which typically produce higher and lower *δ*^13^C signals, respectively [85]. A common finding among these studies is that epiphytic and other algae, not the seagrasses themselves, are a major source of primary production supporting food webs in seagrass habitats [66,86] (although see [87]). For instance, among contemporary studies in the Baltic Sea, invertebrate and vertebrate consumers living in eelgrass habitats had isotopic compositions (low *δ*^13^C, high *δ*^34^S) suggesting that epiphytic algae and other non-seagrass primary producers were the main source of carbon (primary production) supporting the food webs upon which they relied [65,88–91]. The distinctively high *δ*^13^C and low *δ*^34^S values for eels in this study, in contrast, indicate that in these prehistoric seagrass systems primary production from eelgrasses did comparatively more to support some components of local food webs.

In that context, while it is clear that the ecological structure of eelgrass ecosystems, at least those used by eels in the western Baltic, have undergone a considerable shift over time, the underlying drivers of change remain unclear. Factors contributing to these shifts could involve a wide range of variables, including changes in bottom-up (nutrient availability) and top-down (mesograzer intensity and species composition) pressures, which are, in turn, mediated by a range of biotic and abiotic factors, including structural complexity, epiphytic community composition and potential human impacts [80,91–94]. However, the fact this level of subsidy from seagrass primary production (as evidenced by low *δ*^34^S and extremely high *δ*^13^C values) has not frequently been observed in contemporary studies of seagrass food webs (outside of those focusing on herbivorous megafauna specializing in direct consumption of seagrass; for review, see [47]) suggests that something fundamental was different about eelgrass habitats in the distant past. While acknowledging the many potential factors at play, to the extent that anthropogenic eutrophication has been credited with promoting the presence and abundance of epiphytic algae in eelgrass habitats in the region over the past century [18,95], it is possible that the stronger input from eelgrasses to vertebrate food webs in our data reflects eelgrass habitats in an unaltered oligotrophic state, one in which epiphytic algae had a more limited presence than known for the western Baltic today. These data, therefore, offer us an early example of how seagrass ecosystems, at least in this region, may have functioned prior to more recent intensification of human impacts. It is also worth bearing in mind that eels likely recolonized the Baltic Sea during the Littorina Transgression approximately at 6700 cal BCE [4,96] and, while our isotopic evidence postdates this shift by approximately 1200 years, it is possible that marine and estuarine ecosystems in the region were still adjusting ecologically. We believe further research could test our hypothesis about the role of eutrophication and epiphytic algae as a potential driver of the observed eelgrass ecosystem shift. For instance, multivariate analysis of individual amino acid *δ*^13^C values [97,98] shows clear distinctions among algal and seagrass sources. These signals could be examined in past and present fish communities within eelgrass systems to assess whether the functioning of these important coastal habitats has shifted in past centuries or millennia.

### (d) Broader interdisciplinary relevance

Analyses of archaeological samples have an important role to play in future conservation management, at both the ecosystem and species (i.e. multi-ecosystem) levels. Given the globally imperiled nature of eelgrass meadows, and the tremendous range and importance of ecosystem services and tools they are expected to provide for combating climate change (e.g. stabilizing shorelines, capturing and storing carbon, providing biodiversity hotspot refugia), it is imperative that we understand them better [84,99,100]. Likewise, given the critical roles eels play ecologically (e.g. as predators and long-distance nutrient transferers) and in terms of feeding people (contributing to food security) and contributing to the affirmation of a wide range of cultural identities, there is a pressing policy need for research on where and how eels use habitats that have been chronically overlooked [24,27,101]. Much of the research aimed at addressing these challenges has focused on recent timeframes and, while still providing critical insights, these data will often reflect instances in which ecosystems are in a dramatic state flux, either responding to or recovering from human impacts and overexploitation [6]. To the extent that a major goal of ecological research and conservation efforts is to help plan for a future in which humans and non-human animals can coexist in productive, diverse ecosystems (e.g. [1,102]), it stands to reason that basing these plans on information from century- and millennia-scale perspectives will produce more robust frameworks. This means that archaeology will have a valuable role to play in building better strategies to tackle environmental change. The data presented here, which demonstrate the deep antiquity of an important and yet largely unknown link between eels, eelgrass and people, offer a glimpse of this potential. This further highlights the need for increased engagement between archaeology and ecology, which has the potential to open new avenues for understanding ecosystem dynamics over a wider range of ecological and temporal scales, by offering information that cannot be gathered from historical records or contemporary fieldwork.

## Data Availability

All data needed to evaluate the conclusions in the paper are present in the paper and/or the electronic supplementary material [103].
